# Stroke in the adult Qatari population (Q-stroke) a hospital-based retrospective cohort study

**DOI:** 10.1371/journal.pone.0238865

**Published:** 2020-09-21

**Authors:** Yahia Z. Imam, Saadat Kamran, Maher Saqqur, Faisal Ibrahim, Prem Chandra, Jon D. Perkins, Rayaz A. Malik, Naveed Akhtar, Salman Al-Jerdi, Dirk Deleu, Osama Elalamy, Yasir Osman, Gayane Malikyan, Hisham Elkhider, Suha Elmakki, Lubna ElSheikh, Noha Mhjob, Mohamed S. Abdelmoneim, Nima Alkhawad, Ahmed Own, Ashfaq Shuaib

**Affiliations:** 1 Neuroscience Institute, Hamad Medical Corporation, Doha, Qatar; 2 Medical Research Center, Hamad Medical Corporation, Doha, Qatar; 3 Weill Cornell Medicine-Qatar, Doha, Qatar; 4 Division of Neurology, Department of Medicine, University of Alberta, Edmonton, Canada; University of Ioannina School of Medicine, GREECE

## Abstract

**Background:**

Studies assessing the burden of stroke in Qataris are limited. We aim to study stroke in the Qatari population.

**Methods:**

A retrospective review was undertaken of all Qatari adults presenting with stroke to Hamad Medical Corporation over a 5-year period. Descriptive statistics were used to summarize demographic and all other clinical characteristics of the patients. The primary outcome was the incidence of stroke in the Qatari patients. Comparison was made between the sexes.

**Results:**

862 patients were included, with 58.9% being male. The average incidence of stroke over the 5-year period was 92.04 per 100,000 adult Qatari population. The mean age of the cohort was 64.3±14.4 years, (range 19–105 years). The mean age of first ever cerebrovascular event was 63.2±14.5 years. The diagnosis was ischemic stroke in (73.7%), transient ischemic attack in (13.8%), intracerebral hemorrhage (ICH) in (11.6%), subarachnoid hemorrhage in (0.7%) and (0.2%) cerebral venous sinus thrombosis. Small vessel disease was the most common cause of ischemic stroke accounting for (46.5%), followed by large artery atherosclerosis (24.5%). Hypertension (82.7%) and diabetes (71.6%) were particularly prevalent in this cohort. Females were older (65.8±14.1 vs 63.4±14.5 years), had more hypertension and diabetes and more disability or death at 90 days (p<0.05) compared to Qatari males.

**Conclusion:**

Stroke occurs at a significantly lower age in Qataris compared to the western population. This study has uncovered sex differences that need to be studied further.

## Introduction

Stroke poses a global threat and is ranked second to heart disease as the leading cause of death worldwide [1]. Major risk factors for stroke include hypertension, smoking, dyslipidemia, diabetes and obesity. Over the last 40 years, the Arabian Gulf region has transitioned to a more sedentary life style with a parallel rapid increase in the prevalence of major risk factors for stroke [2]. Recent data from the Gulf show that females presenting with atrial fibrillation (AF), a major stroke risk factor, were older, had more co-morbidities and were less likely to undergo cardioversion, although the one year transient ischemic attack (TIA)/stroke rate was comparable to males [3].

Qatar is a peninsula that is located in the Northeastern boarder of the Arabian Peninsula and has a sole land border with Saudi Arabia [4, 5]. It is an oil and gas rich country, but native Qataris represent only 15% of the total population [6]. Qatar is considered to be endemic with obesity, diabetes and cardiovascular disease [7]. Whilst the epidemiology of stroke in Qatar has been well documented [6, 8–10], most studies have focused on the entire population (a population of over 2.7 million) which is disproportionately represented by young able-bodied expatriates working in the country whereas the local Qatari population is only around 300,000.

We have undertaken a detailed assessment of Qatari’s presenting with stroke in relation to clinical and demographic variables, underlying etiology and outcomes. The aim of this work was to describe the demographics and clinical features of stroke in Qataris and to calculate the incidence of stroke in this native population. This study will focus solely on the indigenous Qatari population, which might reflect trends in the region and inform future research.

## Methods

All patients presenting with stroke to Hamad Medical Corporation (the sole provider of acute stroke care in Qatar), from January 2013 thru 2017 were identified. Chart review was undertaken from the 1st of January 2013 to the 31^st^ December 2013, thereafter the Stroke Database (initiated on the 1st of January 2014) was interrogated until 31^st^ December 2017.

Patients were reviewed and included if they were Qatari nationals, ≥18 years of age and had an acute cerebrovascular event (stroke or transient ischemic attack) documented on the discharge, the death summery or labeled as stroke on the Stroke Database. stroke was defined as per the World Health Organization [9] as: ‘‘rapidly developing clinical signs of focal (or global) disturbance of cerebral function, with symptoms lasting 24 hours or longer or leading to death, with no apparent cause other than vascular origin”. Ischemic stroke was defined as rapidly developing clinical symptoms and/or signs of focal disturbance of cerebral function, lasting longer than 24 hours or leading to death, or based on neuroimaging showing an abnormal area matching the clinical picture [8]. TIA was defined as “a brief episode of neurologic dysfunction caused by focal brain or retinal ischemia, with clinical symptoms typically lasting less than one hour, and without evidence of acute infarction” on computed tomography (CT) brain [11]. The stroke was confirmed by documented clinical assessment, CT, magnetic resonance imaging (MRI) scan, or a combination of these [12]. Patients with cerebral infarction, intracerebral hemorrhage (ICH), or subarachnoid hemorrhage (SAH) identified by CT scan or MRI were included. Subdural and extradural hematomas were excluded. Stroke severity was assessed by the National Institute of Health Stroke Scale (NIHSS) [13]. Ischemic strokes were etiologically categorized based on of the Trial of Org 10172 in Acute Stroke Treatment (TOAST) classification [14]. Clinical outcomes were assessed by the modified Rankin Scale (mRS) [15] at 90 days. Additionally, Diabetes Miletus (DM) was defined as per American Diabetes Association criteria [16], dyslipidemia, hypertension, Atrial fibrillation, coronary artery disease and congestive heart failure, were defined as per these respective guidelines [17–20].

### Statistical analysis

Descriptive statistics were used to summarize demographic, clinical, laboratory and other related parameters. Results were reported with the mean and the standard deviation and non-parametric data were presented by the median and the interquartile range (IQR). Categorical data were summarized using frequencies and percentages. Where values were missing the respective percentage was computed based on non-missing values. The primary outcome measure, incidence of stroke, was estimated and presented with the corresponding 95% confidence intervals (CI). Associations between two or more qualitative variables (such as the different risk factors and the types of stroke with sex) were assessed using Chi-square (χ2) test and/or Fisher Exact test as appropriate. Quantitative data (such as age, HbA1c, NIHSS, mRS score etc.) between males and females were analyzed using independent t tests or Mann Whitney U tests where appropriate.

All p values presented were two-tailed, and values <0.05 were considered statistically significant. Data analyses were performed using statistical packages SPSS version 22.0 (SPSS Inc. Chicago, IL).

### Ethical consideration

This study was approved by the Hamad Medical Corporation Medical Research Center, research protocol #15030/15.

## Results

862 patients were included in our study, with 58.9% being male. The mean age of the cohort was 64.3±14.4 years, ranging between 19–105 years. The mean age of first ever cerebrovascular event was 63.2±14.5 years.

The diagnoses were ischemic stroke (IS) in 635(73.7%), TIA in 119 (13.8%), intracerebral hemorrhage (ICH) in 100 (11.6%), subarachnoid hemorrhage (SAH) in 6 (0.7%) and CVST in 2 (0.2%).

TOAST classification was available for 592 of the 635 IS (93.2%). Small vessel disease was the most common cause of IS accounting for 275 (46.5%), followed by large artery atherosclerosis 145 (24.5%), cardio-embolic (CE) 81 (13.7%), other etiologies 21 (3.5%) and cryptogenic 70 (11.8%). Hypertension (82.7%) and diabetes (71.6%) were particularly prevalent in this cohort and AF was present in 104 patients across all the cohort (12.1%) (Table 1).

**Table 1:** Stroke subtype and risk factors by sex.

	Male N = 508	Female N = 354	P-value*
**Mean Age (years)**	63.4±14.5	65.8±14.1	0.02
**Mean NIHSS**	6.1±6.1	6.6±6.3	0.27
**Mean Door-to-needle time (mins)**	52.1±28.4	87±97.5	0.07
**Ischemic Stroke**	369 (72.6%)	266(75.1%)	0.5
**Large vessel disease**	93 (20.6%)	52 (19.7%)	
**Small vessel disease**	164 (35.6%)	111 (36.8%)	
**Cardioembolic**	40 (8.4%)	41 (12.1%)	
**Other etiologies**	10 (4.7%)	11 (3.9%)	
**Unknown etiology**	45 (8.1%)	25 (8.1%)	
**Hemorrhagic stroke**	62 (12.2%)	38 (10.7%)	0.36
**Subarachnoid hemorrhage**	3 (0.6%)	3 (0.8%)	0.4
**TIA**	74 (14.6%)	45 (12.7%)	0.4
**CVST**	0 (0%)	2 (0.6%)	0.4
**†Diabetes Miletus**	350 (68.8%)	269 (75.8%)	0.02
**Hypertension**	412 (80.9%)	303 (85.1%)	0.02
**Dyslipidemia**	255 (50.2%)	177 (49.9%)	0.9
**Coronary heart disease**	157 (30.8%)	114 (32.0%)	0.7
**Heart failure**	34 (6.7%)	27 (7.6%)	0.6
**Atrial fibrillation**	53 (10.4%)	51 (14.4%)	0.08
**Prior stroke/TIA**	114 (22,4%)	73 (20.5%)	0.5
**Thrombolysis**	33 (6.5%)	20 (5.6%)	0.46
**Mean mRS**	1.9±1.9	2.2±2.1	0.10
**Mean mRS at 90 days**	2.1±2.2	2.6±2.2	0.001
**Death at 90 days**	33 (6.5%)	37 (10.4%)	0.04

Key: NIHSS = National Institute of Health Stroke Scale, mRS = modified Rankin Scale TIA = transit ischemic attack, CVST = cerebral venous sinus thrombosis.

*Significant at p<0.05.

On average, patients had strokes which were moderate with a mean NIHSS score of 6.37±5.98. There was no significant difference observed in mean NIHSS between the sexes (Table 1).

Females with stroke were older 65.8±14.1 vs 63.4±14.5 years (p<0.05) (Table 1), had higher rates of diabetes (78.5% vs 88.8%) (p<0.05), and had a trend towards a higher incidence of AF compared to their male counterparts (14.4% vs 10.4% p = 0.08). At 90 days they were more likely to end up with disability (mRS>2) 50.9% vs 39.5% (p<0.005) and a higher mortality (10.4% vs 6.5%) (p = 0.04) (Table 1).

The average incidence of stroke over the 5-year period was 92.04 per 100,000 adult Qatari population. The yearly incidence rate and the percentage with first ever stroke /TIA are shown in (Table 2**)**. The first observed incidence was 81.2/100,000 in 2013 then there was a significant increase in incidence 2014 and 2015 thereafter it has stabilized in 2016 and 2017.

**Table 2:** Estimated stroke incidence per 100,000 per year.

Year	Total population (Qatar)	Estimated Qatari population	Estimated Qatari adult population	Total number of stroke/TIA cases (n)	Incidence (per 100,000) adult population)	95% CI for incidence (lower limit, upper limit)	Number/percentage of first ever stroke/TIA cases
**2013**	2,101,288	252,155	163,900	133	81.2	68.5, 96.2	75 (56.4%)
**2014**	2,172,065	269,648	175,271	183	104.4	90.4, 120.7	113 (61.7%)
**2015**^**$**^	2,404,776	290,000	188,500	187	99.2	86.0, 114.5	158 (84.5%)
**2016**	2,586,776	313,000	203,450	177	87.0	75.1, 100.8	152 (85.9%)
**2017**	2,643,991	322,000	209,300	185	88.4	76.5, 102.1	164 (88.6%)

Key. CI = confidence interval.

$ = census [21].

## Discussion

The demographics of the Qatari cohort presenting with stroke is different to previous reports which included predominantly expatriates. The Qataris in general are older, had a greater number of risk factors and higher prevalence of obesity, diabetes, dyslipidemia and higher prevalence of AF and large artery atherosclerosis compared [6, 22] to non-Qataris.

### Incidence and prevalence

The increase in stroke incidence from 2013 to 2014, 2015 mirrors the establishment of the stroke ward, the stroke database and improvement of patient ascertainment. Thereafter rates have remained static and in keeping with the steady growth of the Qatari population.

In 1997 Hamad et al. [10] reported a crude incidence of stroke to be 75 per 100,000 in the Qatari population. Similarly, El-Hajj et al. [23] reported incidence rates ranging from 15.9–188 per 100,000 when only adults above 18 years of age are included. However, when compared to other local populations in the region (Table 3) [24–31], the incidence of stroke in Qatar within the confines of limited hospital based data appears higher than Saudi Arabia and Kuwait but less than what is reported in Bahrain and Iran. Furthermore, it remains lower than reported in Western cohorts [32–34].

**Table 3:** Stroke incidence in Qatar and the region.

Study	Study period	Country	Population	Number of patients	Crude incidence per 100,000
**Al-Rajeh et al. [24]**	1982–1992	KSA	Saudi nationals	500	43.8
**Al-Rajeh et al. [29]**	1989–1993	KSA	Saudi nationals	488	29.8
**Al-Shenqiti et al. [30]**	2014	KSA	Mixed population¥	164	13.9
**Al-Jishi et al. [28]**	1995	Bahrain	Bahraini nationals	103	57.4
**Al Banna et al. [27]**	2011	Bahrain	Bahraini nationals	521	110
**Abdul-Ghaffar et al. [25]**	1989,1992–1993	Kuwait	Mixed population¥	241	27.6
**Azarpazhooh et al. [31]**	2006–2007	Iran	Iranian population	624	139.0
**Ahangar et al. [26]**	2001–2003	Iran	Iranian population	250	52.0
**Hamad et al. [10]**	2001	Qatar	overall	217	41.0
			Qataris	132	73.0

### Type of stroke

Ischemic stroke was the most prevalent form of stroke, followed by ICH in agreement with data from the region [23] and the world [32–35]. Among those with IS, small vessel disease (SVD) was the most prevalent etiological subtype (36.1%), which is in keeping with regionally reported studies [23]. This is followed by large artery atherosclerotic disease and cardioembolic (CE) etiologies. Previous work has demonstrated that a higher percentage of Qataris have large vessel disease as compared to the younger non-Qatari population [6]. Of note, the etiological subtypes observed here differ significantly from Caucasian populations where CE, large artery atherosclerosis (LAA) or undetermined etiology are more prevalent than SVD [36, 37]. ICH occurred in 11.6% of the cohort, in keeping with data from western countries such as USA, UK and Australia [38] but considerably less than previously reported by Ibrahim et al. (19%) for the mixed population in Qatar predominately composed of South Asians [6].

### Age

Previous data of stroke from Qatar have been generated predominantly from the expatriate population, which is comprised of young working males from the Indian subcontinent and Far East. In the current study Qatari patients with stroke were approximately 6–8 years older than previously reported Qatar based studies (64 versus 56–58 years) [6]. However, this age of stroke is similar to other countries in the region [39–42] but almost a decade younger than that described in Caucasian populations [35, 43].

### Sex

Sex disparities in stroke are well established [44–46], although it is controversial whether sex per se is an independent risk factor for poor prognosis [44, 47]. In our study females were significantly older than males, which is consistent with reports from the region and the West [44, 45, 48]. There was no significant difference in stroke severity as measured by NIHSS or stroke type between the sexes but there was a non-significant trend towards increased AF and CE among female Qataris. Furthermore, females were observed to have a significantly higher prevalence of hypertension, diabetes mellitus, disability at 90 days and mortality, this may be due to sedentary life style with smartphone data showing a lower step/day count for residents in Qatar compared to other countries with women in particular taking 38% less steps [49]. This could be due to availability of household help, unavailability of socially acceptable fitness establishments and the desert climate [50].

Additionally, a recent study [51] hypothesized that maybe poststroke depression and isolation could be a contributing factor for the poor short term outcomes observed in Qatari females, however, this requires more in-depth research.

### Vascular risk factors

Hypertension (82.3%), diabetes (74.4%)and a sedentary life style were very common in this population [52], reflecting the endemic levels of obesity and diabetes found in this part of the world [53]. This contrasts with data from the West, where hypertension and DM are relatively less common, and AF features more often in that older population [54]. The proportion with AF related stroke among the Qatari cohort are more than double that was published for the entire population which is young, male, South Asian predominate [22] but are much less than reported in Western cohorts [55]. Younger age and relatively low utilization of prolonged monitoring, particularly for cryptogenic stroke, [56], maybe contributing to the low incidence of AF reported here.

### Strength and weakness

The major strength of this analysis is the comprehensive search with predefined criteria from a single referral center dealing with the majority of people presenting with stroke in Qatar. A weakness is the retrospective nature of the study with missing data and recall bias.

## Conclusion

The incidence of stroke in Qatari patients has increased compared to 2013 and the proportion of new cases have gone up. Strokes occur a decade earlier than in the western population possibly due to the higher prevalence of vascular risk factors. Additionally, Qatari women with stroke are relatively older, have more atrial fibrillation, diabetes mellitus and hypertension with increased risk of recurrent strokes, disability and higher mortality. Further prospective studies in this population are needed to identify potentially modifiable risk factors and develop effective preventive strategies to limit the increasing incidence of stroke in Qatari’s.

## Supporting information

S1 Data (XLSX)
